# Metropolitan Hospitals Ancient and Modern

**Published:** 1889-01-05

**Authors:** 


*(To be continued.)*

### Metropolitan Hospitals Ancient and Modern.

(By a Medical Superintendent.)

*(Continued from page 175.)*

### II.

Unlike the hospitals, the infirmaries keep well occupied, the reduction, in making the average of beds continuously occupied, being only a-tenth of the certified accommodation, while the hospital average is as much as a fourth of their combined capacity. The most important facts connected with the analysis referred to, which is for the year ending Lady Day, 1884, are those in connexion with the details of expenditure bearing on the several establishments, and explanatory of the cost of each patient, or rather of each permanently-occupied bed. This is found to amount to £35 13*s.* 5*d.* per annum, the maximum being £48 3s. 6*d.*, in the Central London Sick Asylum; and the lowest to £20 11*s.* 8d., at St. Saviour's, Southwark. The extreme difference in the two cases is readily enough accounted for, by the average numbers treated in the two establishments, the first retaining only 197 patients permanently, while St. Saviour's keeps 1,048, although the building was only certified to hold 1,016. Since then, St. Saviour's Infirmary has removed to more salubrious quarters in the vicinity of Dulwich, where the guardians will probably, in the new order of things, find it necessary to increase their former very low average cost. It is now generally understood that the infirmaries, although somewhat under-nursed, are, on the whole, well conducted; they are well officered, the inmates are furnished with everything they absolutely require, and their sanitary arrangements are greatly in advance of most of the general hospitals. Notwithstanding this, one looks in vain in the wards of these palatial establishments for the home comfort, the bright and cheerful surroundings, the bustle and variety caused by a large body of attendants and the freedom of access which the outside world has to the arcana of the interior. On the other hand it is becoming a question whether in aiming at an ideal of perfection we are not shooting a little beyond the mark. It is of little use telling us that it is better to treat a few patients well than a larger number perfunctorily, for we have had abundant evidence that in the large hospitals out of London and in several metropolitan hospitals where nothing can exceed the efficient administration, patients cost 30 per cent. less than they do in others which have nothing to show for it. That this expenditure has been on the increase during the last twenty years and longer, successive reports of the various charities bear witness. It may be traced in all large hospitals, first to the enormous development of the nursing arrangements, higher class and variety in diet of the patients, larger bed space, additional bed and ward furniture, expensive sanitary fittings requiring constant control and supervision, together with a costly assortment of new curative agents and apparatus, all of which must be reckoned with in the future. But for high average cost there is nothing in our metropolitan experience that can at all approach the figures given of the working of the district hospitals for infectious diseases under the control of the Asylums Board. Of the 1,432 beds at the disposal of this body, it appears from the statistics already quoted, that 514, or only a third, were continuously occupied during the year. The analysis also shows that the cost of each bed ranged from a maximum of £615 at the South-Western Hospital, where the permanent accommodation was confined to 11 patients, to a minimum of £154 at another hospital, where the number in daily occupation was 77. The largest daily occupation occurred in the Eastern or Homerton District Asylum where the numbers were 217, detained at the expense of £225 a head, the combined averages of the six hospitals amounting to £283 per occupied bed for the year. These figures are very startling, and can only be explained by the existing necessity of keeping the various establishments fully equipped in the intervals between epidemics, as well as during their occurrence. The Asylums Managers have no easy task in grappling with successive epidemics, but while making every allowances for the difficulties they encounter at every step, one cannot exonerate them from a certain reckless indifference in the manner they spend large sums of money on buildings which for the most part are untenanted. In judging of the management of hospitals by the cost of their occupied beds, we are fully alive to the nature of the objections which may be urged on account of some special features incorporated with the administrative working of individual charities. The out-patient department we have already referred to as forming a stumbling-block in the way of arriving at a correct estimate of cost, and we have given reasons for its omission in our calculations. We are free to admit notwithstanding that there must be a considerable outlay due to this source in most hospitals, less perhaps from the numbers treated than the manner of their treatment. Rooms and attendants are required, a good deal of stationery is consumed, and the drug consumption in the large hospitals is probably two or three times larger than the supply required for the in-patients. Then, again, something must be said in favour of those hospitals which supply all the food the patients may require, instead of throwing the burden of furnishing such necessary articles as tea, sugar, butter, and probably other things, on their friends and relatives.

There is some excuse for hospitals associated with medical schools being somewhat more expensive than others, although we have seen that this is not always the case, and small hospitals are usually conducted on a proportionately more expensive scale than large, on account of the higher prices paid for a smaller supply of food, dressings, and drugs. Nor is it to be lost sight of that the closing of wards at stated intervals has little influence on the permanent machinery of a hospital, any more than it has in the case of the infectious hospitals during the absence of epidemics; but this fact of itself materially increases the average cost of the patients in those hospitals where it is periodically enforced. Apart from the large increase in, and the better entertainment of, the nursing staff, nothing strikes the observer so forcibly as the steady decline in all hospitals in the consumption of alcoholic liquors, and the substitution of milk, ærated waters, and a more varied diet. Malt liquor formed at one time an integral part of the diet in most London hospitals, but it is now either eliminated altogether, or may be exchanged for milk at the option of the medical man or of the patient, to the manifest advantage of the latter. The question of the employment of alcohol, whether as food or medicine, has always been a debatable one among medical men, and it is hardly surprising that hospital committees should continue to look with disfavour on a cause of expenditure, which so many think might be avoided. The managers of hospitals, however, appoint their officers with the fullest confidence in their judgment in questions of medical practice, and the latter would naturally resent any attempt on the part of lay authorities to dictate a line of treatment which they conscientiously felt might prove prejudicial to the patients. Notwithstanding this, and no doubt partly from the marked attention paid to the subject, the consumption of alcoholic stimulants during the last few years has undergone very considerable diminution, both in hospitals, infirmaries, and sick asylums. There is even some reason to fear that the pressure put on the medical officers of these last-named establishments, who hold a less independent position than in such as are supported by voluntary charity, to withhold the administration of stimulants, may not always prove to the advantage of the patients.

But there is another feature with regard to this question and others, which requires further elucidation: In testing comparative expenditure on all articles of hospital consumption, it is very necessary that the prices paid for them should not be left out in the calculation, for it is very possible that one hospital may have to pay twice the amount another does, and consequently gets credited with a larger consumption. The purchase of food and stores opens up a field in which much may be done on behalf of economic administration. Food, liquors, drugs, and dressings are usually supplied—especially food, by open contract, which system, though not free from some disadvantages, is on the whole preferable to other arrangements. Still, contract prices in some of the most simple articles of consumption are often strangely at variance in even the best managed hospitals. The following table represents the results of an inquiry made at six metropolitan hospitals a few years ago, with the view of discovering the practice of each in connexion with prices paid for some of the more important articles of consumption. The price of meat, of which there are generally two or three qualities supplied, was considerably higher a few years ago than it is now.

| Initial Letter | Meat per stone, 8 lbs. | | | Bread per cwt. | Milk per imp gal. | Butter per lb. | Wine per gal. | Brandy per gal. | Whisky per gal. |
|---|---|---|---|---|---|---|---|---|---|
| | s. d. | s. d. | s. d. | s. d. | d. | s. d. | s. d. | s d. | s. d. |
| a. | - - | 4 8 | 6 4 | 12 3 | 10½ | 1 6 | 11 6 | 18 6 | 16 0 |
| b. | - - | 4 8 | 5 4 | 11 1 | 10½ | 1 6 | 9 6 | 21 0 | — |
| c. | - - | 3 6 | 5 2 | 11 1 | 9½ | — | — | — | — |
| d. | 4 4 | 5 4 | 6 0 | 9 11 | 9¼ | 1 3 | 4 5 | 15 9 | 15 |
| e. | 3 4 | 4 4 | 6 8 | 9 11 | 9¼ | 1 2 | 7 10 | 19 0 | — |
| f. | 3 0 | 4 0 | 5 0 | 9 11 | 8 | 1 4 | 4 8 | 12 9 | 16 |

The figures afford a suggestive comment on the cost of maintenance at separate hospitals, but they are cast into the shade with the range of prices which obtain in the metropolitan pauper establishments. The subject is so thoroughly incorporated with questions of administrative economy and its bearing on hospitals that we may be excused for illustrating it further by a few gleanings from the returns of the Local Government Board, detailing the prices paid by the guardians of the several unions for their food supply. The average cost of a pauper in one of the metropolitan workhouses is estimated at £18 18*s.* 8*d.*, the highest cost is £31 15*s.* 3*d.* at St. George's, Mount Street, where there is accommodation for 157 persons only, and the lowest at St. George's, Fulham, where there are, or were, 1,098 beds. The accompanying quotations give the highest and the lowest prices paid by the unions for similar articles of food to those used in the hospitals.

| | Highest Price. | | | Lowest Price. | | |
|---|---|---|---|---|---|---|
| | s. | d. | | s. | d. | |
| Wine per imp. gal.... | 14 | 11 | Hampstead .... | 5 | 3 | St. Pancras. |
| Brandy per imp. gal... | 26 | 0 | Hampstead.... | 16 | 0 | Mile End Old Town. |
| Ale per bar. of 36 gal.. | 52 | 0 | St. Saviour's .. | 25 | 0 | St. Pancras. |
| Porter per bar. of 36 gal. | 38 | 0 | Poplar ......... | 23 | 0 | Camberwell. |
| Bread per cwt... ..... | 14 | 0 | at four Unions | 9 | 4 | at three Unions. |
| Beef per stone 14 lbs... | 10 | 8½ | Westminster .. | 7 | 1 | Fulham. |
| Mutton per st. 14 lbs. | 12 | 6½ | Shoreditch .... | 7 | 8 | Islington. |
| Milk per imp. gal .... | 1 | 0 | at three Unions | 0 | 9 | Bethnal Green. |
| Butter per cwt......... | 154 | 0 | Fulham ...... | 72 | 0 | St. Giles, Blmsbry. |
| Tea per lb............ | 2 | 0 | Hampstead.... | 1 | 1½ | Chelsea. |

The cost of some minor matters, not referred to in the above list, is exceedingly bewildering. Why twelve pounds of candles should cost 12*s.* at Lewisham and only 6*d.* at Mile End is by no means clear, and how the obsequies of a pauper can be conducted for the modest sum of 11*s.* 6*d.* in Bethnal Green, while it takes 49*s.* 2*d.* to perform the same ceremony in the neighbouring district of Stepney is a puzzle to the uninitiated. On the whole, the hospitals appear rather to have the advantage of the workhouses as regards economy in food, while the discrepancies in prices in both, point to an evil which is remediable, and being so, might be the means of vastly reducing expenditure. Neither hospital committees nor Boards of Guardians can lay claim to an astute knowledge of the quality or market value of the perishable and other commodities in daily use in their respective establishments, and, failing this, they fall back on the safe premise that to obtain a good article they must pay a fair price for it. The proposition is good in the abstract, nor is it in any way compulsory to accept the lowest tenders, but the varieties and fluctuations in the prices of food, sick-room appliances, drugs, and dressings, require more consideration than is usually given them, and there are many things outside the system of contract altogether, requiring both knowledge and care in their selection. It would be infinitely more satisfactory if the task of providing charitable and other establishments with articles in daily use were delegated to a committee of experts, who from a central store could receive and issue to each establishment daily what is required without disturbing its autonomy.

*(To be continued.)*

